# An axiomatic characterization of the Borda mean rule

**DOI:** 10.1007/s00355-018-1167-8

**Published:** 2018-12-17

**Authors:** Florian Brandl, Dominik Peters

**Affiliations:** 1 Department of Informatics, Technical University of Munich, Munich, Germany; 2 Department of Computer Science, University of Oxford, Oxford, UK

## Abstract

A social dichotomy function maps a collection of weak orders to a set of dichotomous weak orders. Every dichotomous weak order partitions the set of alternatives into approved alternatives and disapproved alternatives. The Borda mean rule returns all dichotomous weak orders that approve all alternatives with above-average Borda score and disapprove alternatives with below-average Borda score. We show that the Borda mean rule is the unique social dichotomy function satisfying neutrality, reinforcement, faithfulness, and the quasi-Condorcet property. Our result holds for all domains of weak orders that are sufficiently rich, including the domain of all linear orders and the domain of all weak orders.

## Introduction

The typical objective of social choice is to choose the best alternatives based on voters’ preferences. Preferences are given as a weak order for each voter. Using this information, *social choice functions* choose a set of winning alternatives. Suppose instead that the goal is to split the alternatives into good alternatives and bad alternatives with the *separation* between both sets being as large as possible. Duddy et al. (2014) note that social choice functions, viewed as producing a partition into winning and non-winning alternatives, are not the right tool for this task. For example, consider a class of students that is to be divided into beginners and advanced learners based on how they are ranked by teachers. The goal is to form two groups of students such that the differences in skill level within each group are as small as possible. If all teachers agree on their top-ranked student, any reasonable social choice function would uniquely choose the unanimously top-ranked student. Hence, the group of advanced learners would consist of only this one student; all other students would be put into the beginners group. In our example this is likely to be an undesired result, since the differences in skill within the beginners group would be barely reduced compared to the entire class.

Thus, we need to drop some of the properties that seem appealing for social choice functions. A more suitable tool for our task are *social dichotomy functions* (Duddy et al. 2014), which yield ordered 2-partitions of the alternatives. We interpret ordered 2-partitions as having the approved alternatives in the first set and the disapprovedalternatives in the second set. In contrast to selecting the best alternatives, there is inherent symmetry in the problem of finding a good separation; in particular, social dichotomy functions should usually satisfy *reversal symmetry*: if all of the inputpreferences are reversed, then the output will also be reversed, so that approved and disapproved alternatives switch place.

Social dichotomy functions are appealing as a tool for summarizingpreference information: a group can compile their preferences to obtain a single binaryclassification into promising and unpromising alternatives. In general, the input and output of a social dichotomy function may express any kind of structure on the set of alternatives; it need not suggest a better–worse relationship. For example, suppose a number of experts has different assessments on how political parties should be ordered on the left–right spectrum. Based on this information, a social dichotomy function can be used to group parties into left-wing and right-wing.^1^

**Fig. 1:**
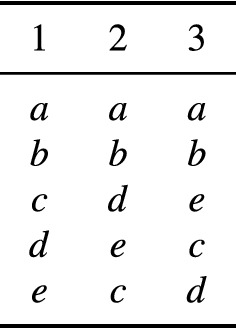
A preference profile for three voters and five alternatives. Each column depicts the preferences of one voter whose preference ranking is listed from top to bottom. The symmetric Borda scores of *a* and *b* are 12 and 6, respectively; each of *c*, *d*, and *e* has a symmetric Borda score of $-\,6$. Hence, the Borda mean rule returns the dichotomous weak order that approves *a* and *b* and disapproves *c*, *d*, and *e*. Note that even though *b* is Pareto dominated by *a*, *b* is approved

We consider a social dichotomy function that is based on *Borda scores*. A *preference profile* specifies for each voter a weak order on an alternative set *A*. Given a preference profile, the (symmetric) Borda score of an alternative *a* is obtained as follows: *a* gains a point for each voter *i* and each alternative *b* such that $a\succ _i b$; *a* loses a point if $b\succ _i a$; no points are assigned if $a\sim _i b$. Hence, the average Borda score is 0. *Borda’s rule* is the social choice function that returns the alternatives with maximum Borda score. The social dichotomy function that we consider in this paper is the *Borda mean rule* which outputs all dichotomous weak orders in which all alternatives with above-average Borda score are approved, and all alternatives with below-average Borda score are disapproved. If there are alternatives with precisely average Borda score, then the rule returns several orders with all ways of breaking the ties. This rule was defined by Duddy et al. (2014) and further discussed by Duddy et al. (2016, Section 5) and Zwicker (2018). Notice that the Borda mean rule satisfies reversal symmetry. An example illustrating the Borda mean rule is given in Fig. 1.

Reversal symmetry (similarly defined) is also a natural property for *socialpreference functions*, which return a set of *linear* orders of the alternatives. We will see that social dichotomy functions are more closely related to social preferencefunctions than to social choice functions. Kemeny’s rule (Kemeny 1959) is an example of a social preference function that has been very influential in social choice theory. Young (1995) predicted that “the time will come when it is considered a standard tool for political and group decision making”. Given a preference profile, the rule assigns to each possible weak order $\succcurlyeq$ a *Kemeny score*: the order gains a point for each voter *i* and each pair of alternatives $a,b\in A$ such that $a\succ b$ and $a\succ _i b$; the order loses a point if $b \succ _i a$; no points are assigned if $a \sim _i b$. Thus, the Kemeny score of$\succcurlyeq$ indicates how much pairwise “agreement” there is between the output order $\succcurlyeq$ and the input preferences. Kemeny’s rule returns the set of all *linear orders* with maximum Kemeny score.

Zwicker (2018) introduced the idea of using Kemeny scores to define aggregation rules for other output types. For example, suppose we maximize the Kemeny score over the family of relations $\{x\} \succ A{\setminus }\{x\}$ that have a unique most-preferred element and that are indifferent between all other alternatives. This yields precisely the relations whose most-preferred element is a winner of *Borda’s rule*. In his paper, Zwicker (2018) proposed the *k*-*Kemeny rule* which returns the *k*-chotomous weak orders of highest Kemeny score; a weak order $\succcurlyeq$ is called *k*-*chotomous* if its induced indifference relation $\sim$ partitions *A* into at most *k* indifference classes.^2^ 2-chotomous orders are usually called *dichotomous*; these are the orders that partition the alternatives into a set of *approved* and a set of *disapproved* alternatives. Hence, the 2-Kemeny rule is a social dichotomy function. Duddy et al. (2014) showed that the 2-Kemeny rule is identical to the *Borda mean rule*. This equivalent definition of the Borda mean rule suggests that it is a good tool for finding dichotomies that maximize the separation between the set of approved and the set of disapproved alternatives.

Social choice theory abounds with proposals for voting rules; which of them should we use, and in which contexts? Axiomatic characterizations provide some of the strongest reasons in favor of using certain rules. For example, Kemeny’s rule is largely seen as a very attractive social preference function because of its characterization by Young and Levenglick (1978) [though there are other reasons, such as the rule’s interpretation as a maximum likelihood estimate (Young 1988)]. In this paper, we present an axiomatic characterization of the Borda mean rule, using the same axioms as the characterization of Kemeny’s rule by Young and Levenglick (1978). Thus, the above argument in favor of Kemeny’s rule applies just as well to the Borda mean rule, hopefully establishing its place as a very natural social dichotomy function. In formal terms, our result is that the Borda mean rule is the unique social dichotomy function satisfying neutrality, reinforcement, faithfulness, and the quasi-Condorcet property.^3^ Our proof follows a similar structure to Young’s (1974a) characterization of Borda’s rule. In particular, we also use linear algebra and exploit the orthogonal decomposition of weighted tournaments popularized in social choice theory by Zwicker (1991).^4^ In contrast to Young and Levenglick (1978), we do not need any convex separation theorems.

Most of our axioms are commonly used, including the uncontroversial axioms of neutrality (requiring that all alternatives are treated equally) and faithfulness(requiring sensible behavior in single-voter situations). Reinforcement (often known as*consistency*) is the workhorse of many axiomatic characterizations in social choice. It is a variable-electorate axiom which requires that if the same dichotomy is selected in two disjoint profiles, then it is still selected if we merge the two profiles into one. Reinforcement is typically satisfied by rules which maximize a sum of the “scores” that each voter assigns to a potential output. The most specialized axiom in ourcollection is the quasi-Condorcet property, introduced by Young and Levenglick (1978) and also used by Barthélemy and Janowitz (1991). It requires that a “dummy alternative” (one that is tied with every other alternative in a pairwise majoritycomparison) can move around freely within the output relation. (We give a formaldefinition below.) The quasi-Condorcet property is stronger than the *cancellation axiom* and thus, in conjunction with reinforcement, implies that the output can only depend on the weighted majority relation (see Lemma 2). None of the four axioms in our collection can be dropped without the characterization result breaking down (see Sect. 8).

Our result applies to various possible input types. In particular, it applies when votes are given by linear orders, or when they are given by arbitrary weak orders. It also applies to *j*-chotomous weak orders whenever $j \geqslant 3$; thus, our result characterizes Zwicker’s (2018) (*j*, 2)-*Kemeny rule* for each $j\geqslant 3$, which is the Borda mean rule as applied to profiles of *j*-chotomous weak orders. More generally, our proof works whenever the domain of allowed preference orders forms a *McGarvey domain*, that is, whenever every possible weighted majority tournament (with only even weights or only odd weights) can be induced by a profile using such orders. The domains of linear orders, of weak orders, and of *j*-chotomous orders ($j\geqslant 3$) are McGarvey domains. In contrast, the domain of dichotomous orders is not a McGarvey domain, and our proof does not apply to this domain.

## Related work

Duddy et al. (2016) study a setting in which every voter holds a binary evaluation of the alternatives or, equivalently, a dichotomous weak order. A *binary aggregation function* maps the voters’ binary evaluations to an ordered 3-partition of approved, tied, and disapproved alternatives. Thus, the output of a binary aggregation function assigns to each alternative one of the values $+\,1$, 0, or $-\,1$. Duddy et al. (2016) propose the *mean rule*, which assigns $+\,1$ to all alternatives with above-average approval score, assigns 0 to alternatives whose approval score is exactly average, and assigns $-\,1$ to all alternatives with below-average approval score. They explain that the mean rule can be used in *judgement aggregation* for certain agendas, and connect the mean rule with the scoring rules for judgement aggregation introduced by Dietrich (2014). Further, Duddy et al. (2016) prove that the mean rule is the only binary aggregation function satisfying axioms called neutrality, consistency, cancellation, and faithfulness. Their notion of consistency is a version of Smith’s (1973) axiom of *separability*: if an alternative is approved by one electorate and either approved or ranked as tied by another disjoint electorate, then it is approved by the union of both electorates (and analogously for disapproved alternatives). While the mean rule is very closely related to the Borda mean rule (when evaluated on profiles of dichotomous weak orders), the formal setting of Duddy et al. (2016) differs significantly from ours. In particular, our characterization result is logically independent from theirs, since our reinforcement axiom neither implies nor is implied by their consistency axiom. Further, our proof does not work for the case where voters are only allowed to submit dichotomous weak orders, and their proof does not apply to the kinds of input types that we study.

Since social dichotomy functions can be viewed as returning a set of multiplewinners, the recent literature on *multiwinner voting rules* is related (for a survey, see Faliszewski et al. 2017a). Voting rules in that setting return a committee of *k*alternatives, where *k* is fixed. Examples include the *k*-Borda rule (which returns the *k* alternatives with highest Borda score, see Debord 1992), as well asChamberlin and Courant’s (1983) rule and Monroe’s (1995) rule, which aim forcommittees providing proportional representation. Note that, in contrast, the definition of a social dichotomy function does not impose any cardinality constraint on the set of approved alternatives. Indeed, multiwinner rules typically do not satisfy reversal symmetry. Axiomatic characterizations of multiwinner rules using consistency-type axioms are provided by Skowron et al. (2016) for linear order preferences and by Lackner and Skowron (2018) for approval preferences. The *k*-Borda rule wascharacterized by Debord (1992); his result is close to ours, but simpler to prove due to the cardinality constraint on the set of winners. The *k*-Borda rule can be equivalently defined as the rule that returns the Kemeny score-optimal dichotomous orders with exactly *k* approved alternatives.

Recently, there has also been some discussion of multiwinner voting rules with a *variable* number of winners. The Borda mean rule is an example of such a rule. Kilgour (2016) reviews several such rules for the case of approval (dichotomous) preferences, and Faliszewski et al. (2017b) study their computational complexity.

A recent paper by Lang et al. (2016) proposes several schemes of rules that can be used to aggregate preferences into an arbitrary structure. For example, they propose a Kemeny scheme that can be used to find an aggregate ranking, or a committee, or a single winner, or an ordered committee, etc. Applying their Kemeny scheme to the output type of a dichotomy (i.e., an ordered partition into two pieces) yields the Borda mean rule. They also propose two other schemes that, specialized to dichotomies, yield different rules. The first is based on minimizing a Hamming distance and yields the *Copeland mean rule*, which approves an alternative whenever its Copeland score is above-average. The second generalizes the *Ranked Pairs rule* due to Tideman (1987), and yields a Ranked Pairs rule for dichotomies.

Many characterizations of Borda’s rule as a social choice function, and of scoring rules more generally, are available (for a survey, see Chebotarev and Shamis 1998). Young (1974a) gave the first characterization of Borda’s rule using reinforcement. Hansson and Sahlquist (1976) gave an alternative proof that does not use linear algebra. Young (1975) characterized the class of all scoring rules, and identified Borda’s rule among them by adding an additional axiom (cancellation). Smith (1973) independently found a characterization of scoring rules as social welfare functions; Young (1974b) gave an alternative proof of that result.

The Borda mean rule is also related to Nanson’s rule, which, in order to determine a winner, repeatedly eliminates all alternatives with below-average Borda score (Nanson 1882; Niou 1987). The Borda mean rule is, in a sense, the result of stopping Nanson’s procedure after its first round.

The quasi-Condorcet property, a key axiom in our characterization, was introduced by Young and Levenglick (1978) for characterizing Kemeny’s rule. The axiom also proved useful in the literature about the *median procedure* for aggregating other kinds of data structures, such as for median semilattices (Barthélemy and Janowitz 1991) and median graphs (McMorris et al. 2000). Nehring and Pivato (2018) characterized the median procedure in judgement aggregation using reinforcement and a property called supermajority efficiency. Their result also yields an alternative characterization of Kemeny’s rule.

## Definitions

We use ${\mathbb {N}} = \{1,2,\ldots \}$ to denote an infinite set of potential voters. Let *A* be a finite set of alternatives, where $|A| = m$. The preferences of an agent $i\in {\mathbb {N}}$ are given by a binary relation ${\succcurlyeq _i}\subseteq A\times A$ which is complete and transitive; such a relation is called a *weak order*. We will write $a\succ _i b$ if $a\succcurlyeq _i b$ but $b\not \succcurlyeq _i a$, and $a\sim _i b$ if both $a\succcurlyeq _i b$ and $b\succcurlyeq _i a$. The *reverse*$-{\succcurlyeq }$ of a weak order $\succcurlyeq$ is defined by $(a,b) \in -{\succcurlyeq }$ if and only if $(b,a) \in {\succcurlyeq }$. If $\sigma$ is a permutation of *A*, we can naturally define the relation $\sigma ({\succcurlyeq }) = \{ (\sigma (a), \sigma (b)) :(a,b) \in {\succcurlyeq } \}$, and extend this definition to sets and profiles of weak orders.

A weak order $\succcurlyeq$ is called a *linear order* if it is antisymmetric, so that $a\sim b$ only if $a = b$. A weak order $\succcurlyeq$ is *dichotomous* if there is a partition $(A_1, A_2)$ of *A* into two subsets such that $a \succ b$ if and only if $a\in A_1$ and $b\in A_2$. We allow one of $A_1$ and $A_2$ to be empty, in which case ${\succcurlyeq } = A\times A$ is complete indifference. Equivalently, an order is dichotomous if and only if there are no three alternatives $a,b,c\in A$ with $a\succ b \succ c$. We will write ${\mathscr {R}}(A)$ for the set of all weak orders over *A*, ${\mathscr {L}}(A)$ for the set of all linear orders over *A*, and ${\mathscr {R}}_2(A)$ for the set of all dichotomous weak orders over *A*. When the set *A* is clear from the context, we write ${\mathscr {R}}$, ${\mathscr {L}}$, and ${\mathscr {R}}_2$, respectively.

An *electorateN* is a finite and non-empty subset of ${\mathbb {N}}$. The set of all electorates is denoted by ${\mathscr {F}}({\mathbb {N}})$. A *(preference) profile*$P \in {\mathscr {R}}^N$ on electorate *N* is a function assigning a weak order to each voter in *N*. The preferences of voter *i* in profile *P* are then denoted by $\succcurlyeq _i$.

A *domain*${\mathscr {D}}\subseteq {\mathscr {R}}$ is a set of weak orders that the voters are allowed to submit. Typical choices for ${\mathscr {D}}$ will be ${\mathscr {R}}$ or ${\mathscr {L}}$. A *social dichotomy functionf* is a map from the set of all profiles in ${\mathscr {D}}^N$ for some $N\in {\mathscr {F}}({\mathbb {N}})$ to non-empty subsets of ${\mathscr {R}}_2$, so that $f(P) \subseteq {\mathscr {R}}_2$ for all profiles *P*.^5^ We denote by $-\,f$ the social dichotomy function that returns the reverse of the weak orders returned by *f*; thus, $(-f)(P) = -(f(P))$, for all profiles *P*.

Given a profile $P\in {\mathscr {R}}^N$ on the electorate *N* we denote by *T*(*P*) the matrix of majority margins induced by *P*, i.e., for all $a,b\in A$,

$$\begin{aligned}{}[T(P)]_{ab} = |\{i \in N :a \succ _i b \}| - |\{ i \in N :b \succ _i a\}|. \end{aligned}$$

Note that *T*(*P*) is a skew-symmetric $m\times m$ matrix with zeros on the main diagonal (since $[T(P)]_{ab} = -[T(P)]_{ba}$).^6^ When ${\mathscr {P}}$ is a set of profiles, $T({\mathscr {P}}) = \{T(P):P\in {\mathscr {P}}\}$ is the set of matrices induced by profiles in ${\mathscr {P}}$. We can interpret *T*(*P*) as a *weighted tournament* whose vertices are given by the alternatives; there is an arc from *a* to *b* if and only if $[T(P)]_{ab} > 0$, and the arc is labelled by $[T(P)]_{ab}$. For a permutation $\sigma$ of *A*, we denote by $\sigma (T)$ the weighted tournament whose vertices are relabelled according to $\sigma$, so that the arc from $\sigma (a)$ to $\sigma (b)$ has weight $[T(P)]_{ab}$ for all $a,b\in A$.

The domains we consider are required to be sufficiently rich, a notion defined in terms of the weighted tournaments they induce. A domain ${\mathscr {D}}$ is a *McGarvey domain* if every weighted tournament that can be induced by a preference profile of linear orders can be induced by a profile on the domain. Formally,

McGarvey domain $$\begin{aligned} T(\{P\in {\mathscr {L}}^N:N\in {\mathscr {F}}({\mathbb {N}})\}) \subseteq T(\{P\in {\mathscr {D}}^N:N\in {\mathscr {F}}({\mathbb {N}})\}). \end{aligned}$$

It has been shown by Debord (1987) that the set of linear orders can induce exactly those integral weighted tournaments whose off-diagonal entries all have the same parity. Hence, a domain is a McGarvey domain if and only if it can induce all those weighted tournaments.^7^ Examples of McGarvey domains are ${\mathscr {L}}$, ${\mathscr {R}}$, and, for each $j\geqslant 3$, the set of all *j*-chotomous weak orders on *A*. Examples of domains that are not McGarvey domains are the set of dichotomous weak orders ${\mathscr {R}}_2$, and the set of weak orders that are single-peaked with respect to some fixed axis: every profile from either of these domains has a transitive majority relation, so they cannot be McGarvey domains.

The (symmetric) *Borda score*$\beta _P(a)$ of an alternative $a\in A$ in a profile *P* is given by

$$\begin{aligned} \beta _P(a) := \sum _{b\in A{\setminus }\{a\}} [T(P)]_{ab}, \end{aligned}$$

the net weighted out-degree of *a* in *T*(*P*). This definition of Borda scores makes sense for profiles of arbitrary weak orders. For the case of linear orders, it is easy to see that $\beta _P$, thus defined, is a positive affine transformation of the Borda scores as defined through the usual positional scoring vector $(m-1, m-2, \dots , 1, 0)$; indeed, the positional Borda score of *a* is $\beta _P(a)/2 + |N|(m-1)/2$. Thus, for example, the same alternatives are Borda winners for either definition of Borda scores.^8^ Note that, because the majority margins are skew-symmetric, we have $\sum _{a\in A} \beta _P(a) = 0$, and so the average (symmetric) Borda score of the alternatives is always 0, which makes it convenient to deal with symmetric Borda scores.

## Borda mean rule

As we have mentioned in the introduction, there are several equivalent ways of defining the Borda mean rule. The most straightforward definition uses the average Borda score directly. The Borda mean rule $BM$ is the social dichotomy function with

$$\begin{aligned} BM (P) = \Big \{ {\succcurlyeq } \in {\mathscr {R}}_2 :a \succ b \text { for all }a,b\in A\text { with }\beta _P(a) > 0\text { and }\beta _P(b) < 0 \Big \} \end{aligned}$$

for every preference profile $P\in {\mathscr {D}}^N$ on any electorate $N \in {\mathscr {F}}({\mathbb {N}})$. Thus, the Borda mean rule returns all dichotomous weak orders where alternatives with above-average Borda score are placed in the upper indifference class and alternatives with below-average Borda score are placed in the lower indifference class. This implies that the Borda mean rule returns exactly $2^k$ dichotomous weak orders, where *k* is the number of alternatives with exactly average Borda score. These alternatives appear in all possible ways in the upper and in the lower indifference class.

In the framework of Zwicker (2018), the Borda mean rule is obtained as a special case of Kemeny’s rule with dichotomous output. Precisely, the Borda mean rule is the rule returning the dichotomous weak orders of maximum Kemeny score (as defined in Sect. 1):

$$\begin{aligned} BM (P) = \mathop {\hbox {arg max}}\limits _{{\succcurlyeq }\in {\mathscr {R}}_2} \sum _{x\succ y} [T(P)]_{xy}. \end{aligned}$$

Hence, any ${\succcurlyeq }\in BM (P)$ maximizes the pairwise agreement with the voters’preferences among all dichotomous weak orders.

It can be observed from the definition that the Borda mean rule only depends on the pairwise majority margins and hence on the weighted tournament induced by a preference profile.^9^ This property will play an important role in our characterization.

An interesting property of the Borda mean rule is that it always approves Condorcet winners and always disapproves Condorcet losers, provided they exist. This can be seen by recalling that if *a* is the Condorcet winner in a profile *P*, then $\beta _P(a) > 0$ from the definition of $\beta _P$, and similarly $\beta _P(b) < 0$ if *b* is the Condorcet loser. We can argue alternatively that the Kemeny score of a dichotomy $\succcurlyeq$ strictly improves if we move the Condorcet winner from the lower to the upper indifference class.

**Fig. 2:**
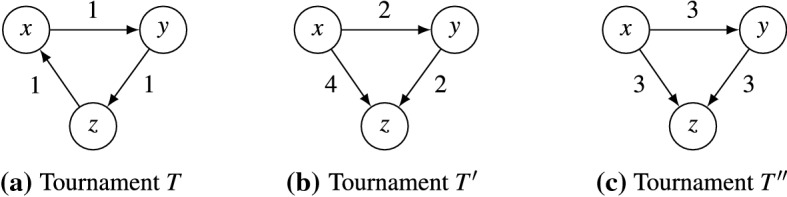
Examples for the Borda mean rule. The weight of an arc denotes the majority margin between the two adjacent alternatives

To help us understand the Borda mean rule, we discuss its behavior for the weighted tournaments given in Fig. 2. Let *P* be the profile on electorate $N = \{1,2,3\}$ with $x \succ _1 y \succ _1 z$, and $y \succ _2 z \succ _2 x$, and $z \succ _3 x \succ _3 y$. This profile induces the tournament $T = T(P)$, in which all alternatives have Borda score 0 and so we have $BM (P) = {\mathscr {R}}_2$. Next, consider the profile $P'$ on electorate $N' = \{4,5,6,7\}$, where $x \succ _i y \succ _i z$ for $i\in \{4,5\}$, and $x \succ _6 z \succ _6 y$ and $y \succ _7 x \succ _7 z$. Also, write $P'' = P \cup P'$ for the profile obtained by merging *P* and $P'$. These profiles induce $T' = T(P')$ and $T'' = T(P'')$. In both $T'$ and $T'' = T + T'$ the Borda scores are $6, 0, -6$ for *x*, *y*, *z*, respectively. Hence $BM (P') = BM (P'') = \{\{x,y\}\succ \{z\}, \{x\}\succ \{y,z\}\}$.

## Axioms

Duddy et al. (2014) argue that social dichotomy functions should satisfy *reversal symmetry*: if all voters reverse their preferences, then the approved set becomes the disapproved set and *vice versa*. Formally, a social dichotomy function satisfies reversal symmetry if

Reversal symmetry $$\begin{aligned} f(- P) = -{f(P)}\quad \text {for all } P\in {\mathscr {D}}^N \text { and } N\in {\mathscr {F}}({\mathbb {N}}). \end{aligned}$$

While the Borda mean rule satisfies reversal symmetry, we do not impose this axiom for our characterization (it must therefore be implied by our other axioms). Instead, we use the same four axioms that also feature in Young and Levenglick’s (1978) characterization of Kemeny’s rule. First, we require social dichotomy functions to satisfy *neutrality*: renaming the alternatives in a preference profile leads to the same renaming in the output relations. Neutrality thus prescribes that a social dichotomy function is symmetric with respect to the alternatives and prevents it from being biased towards certain alternatives. Let $\varPi (A)$ denote the set of all permutations on *A*. Then, a social dichotomy function *f* satisfies neutrality if

Neutrality $$\begin{aligned} f(\sigma (P)) = \sigma (f(P))\quad \text {for all } P\in {\mathscr {D}}^N, N\in {\mathscr {F}}({\mathbb {N}}) \text {, and } \sigma \in \varPi (A). \end{aligned}$$

When dealing with variable electorates, it seems reasonable to require that the rankings that are returned for two disjoint electorates should be precisely those that are returned when the electorates are merged. If the output for two electorates does not intersect, the condition says nothing. This is known as *reinforcement*. A social dichotomy function *f* satisfies reinforcement if

Reinforcement $$\begin{aligned}&f(P)\cap f(P') \ne \varnothing \!\text { implies } f(P)\cap \!f(P') \!= f(P\cup P') \text {for all } P\!\in {\mathscr {D}}^N \text { and } P'\!\in \!{\mathscr {D}}^{N'}\\&\quad \text {with } N\cap N' = \varnothing . \end{aligned}$$

Notice that reinforcement is agnostic about the type of output. It may be defined in the same way for every kind of aggregation function, such as social choice functions (which return a subset of alternatives) or social preference functions (which return a set of linear orders of the alternatives). Reinforcement was introduced by Young (1974a, 1975) (he called it *consistency*) to characterize scoring rules; the axiom is related to *separability* introduced by Smith (1973) (now often also called consistency) for social welfare functions.

Next, we consider an axiom that specifies how to deal with “dummy”alternatives that are independent from the others in the sense that they are tied with every other alternative in a pairwise majority comparison. Formally, an alternative $x\in A$ is a *dummy* in a profile *P* if $[T(P)]_{xy} = 0$ for all $y\in A$. The *quasi-Condorcetproperty* asserts that dummy alternatives can be placed arbitrarily in the output relation.Intuitively, this axiom claims that we have no relevant information about theappropriate placement of dummy alternatives in the output. For a set $B\subseteq A$, we say that two dichotomous weak orders $\succcurlyeq$ and $\succcurlyeq '$*agree on B* if for all $x,y\in B$, we have $x \succcurlyeq y$ if and only if $x \succcurlyeq ' y$. Then, a social dichotomy function *f* satisfies the quasi-Condorcet property if

Quasi-Condorcet property $$\begin{aligned}&{\succcurlyeq } \in f(P) \text { if and only if } {\succcurlyeq '} \in f(P)\quad \text {whenever }\succcurlyeq \text { and }\succcurlyeq '\text { agree on }A {\setminus } D, \\&\text {where } D = \{x\in A:[T(P)]_{xy} = 0\text { for all } y\in A\} \text {, for all } P\in {\mathscr {D}}^N \text { and } N\in {\mathscr {F}}({\mathbb {N}}). \end{aligned}$$

**Fig. 3:**
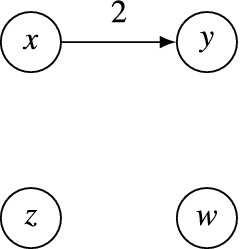
A tournament *T* with two dummy alternatives *z* and *w*. The arcs not drawn have weight 0

Since it is the least intuitive of our axioms, we give an example that illustrates the consequences of the quasi-Condorcet property. Consider a preference profile *P* on electorate $N = \{1,2\}$ where $x\succ _1 y \succ _1 z \succ _1 w$ and $w\succ _2 z \succ _2 x \succ _2 y$. This profile induces the weighted tournament $T = T(P)$ depicted in Fig. 3. Note that *z* and *w* are dummies in *T*, so $D = \{z,w\}$. If ${\succcurlyeq }\in f(P)$ with $\{x,z,w\}\mathrel {\succ }\{y\}$, then the quasi-Condorcet property implies that the following three dichotomous weak orders are also contained in *f*(*P*):

$$\begin{aligned} \{x\}\succ '\{y,z,w\} \quad \{x,z\}\succ '\{y,w\} \quad \{x,w\}\succ '\{y,z\} \end{aligned}$$

In Corollary 1, we show that in conjunction with the other axioms, the quasi-Condorcet property implies that not only dummy alternatives can be placed arbitrarily in the output relation, but that this applies to all alternatives with Borda score 0.

The quasi-Condorcet property is a strengthening of the *cancellation* axiom, which requires that all dichotomies are returned whenever all majority margins are zero. Formally, *f* satisfies cancellation if

Cancellation $$\begin{aligned} f(P) = {\mathscr {R}}_2 \quad \text {for all } P\in {\mathscr {D}}^N\text { and } N\in {\mathscr {F}}({\mathbb {N}}) \text { with } [T(P)]_{xy} = 0 \text { for all } x,y\in A. \end{aligned}$$

To see that the quasi-Condorcet property implies cancellation, observe that whenever all majority margins are zero in some profile *P*, every alternative is a dummy. Thus, every two dichotomous weak orders agree on the (empty) set of alternatives that are not dummies. Hence, the quasi-Condorcet property requires that $f(P) = {\mathscr {R}}_2$.

Our axioms so far do not rule out the trivial social dichotomy function which always returns all dichotomies. We need an axiom that prescribes some degree of correlation of the voters’ preferences with the aggregated dichotomies. An arguably minimal axiom of this nature is *faithfulness*, which requires that whenever the electorate consists of just one voter, the aggregated preferences should not contradict that voter’s preferences. Formally, a social dichotomy function *f* satisfies faithfulness if

Faithfulness $$\begin{aligned} {\succ _i}\subseteq {\succcurlyeq } \quad \text {for all } {\succcurlyeq }\in f(P)\text {, } P\in {\mathscr {D}}^{\{i\}} \text {, and } i\in {\mathbb {N}}. \end{aligned}$$

Thus, if *P* is a profile with only one voter who strictly prefers *a* to *b*, then *f*(*P*) cannot contain any dichotomy which approves *b* but disapproves *a*. As an example, if *P* is a profile containing only the vote of voter *i*, and $\succcurlyeq _i$ is the linear order $a \succ _i b \succ _i c \succ _i d$, then faithfulness requires that *f*(*P*) contains only dichotomous orders that approve an initial segment of $\succcurlyeq _i$ (i.e., an upper contour set of some alternative). Thus $\{a,b\}\succ \{c,d\}$ may be a member of *f*(*P*), but $\{a,d\} \succ \{b,c\}$ cannot be a member of *f*(*P*).

Faithfulness is a weak axiom. It is even weaker than the following (weak) Pareto axiom: whenever a profile $$P\in {\mathscr {D}}^N$$, $$N\in {\mathscr {F}}({\mathbb {N}})$$, is such that $$a \succ _i b$$ for all $$i\in N$$, then $$a \succcurlyeq b$$ for all $${\succcurlyeq } \in f(P)$$. In the proof of our characterization, the faithfulness axiom is invoked only once, to rule out the two social dichotomy functions that satisfy the remaining axioms: first, the trivial social dichotomy function $$ TRIV $$, which always returns all dichotomous weak orders, and second, the reverse Borda mean rule $$- BM $$.

## The linear algebra of weighted tournaments

For our characterization, it will be useful to understand the structure of weighted tournaments better, and so we give a brief introduction to their linear algebra. Let *V* be the vector space of rational-valued skew-symmetric $m\times m$ matrices (or, equivalently, of weighted tournaments with rational arc-weights). Note that the dimension $\dim V$ of *V* is $$\left( {\begin{array}{c}m\\ 2\end{array}}\right) $$. In this context, we write $0\in V$ to refer to the $m\times m$ matrix all of whose entries are 0. Identifying a skew-symmetric matrix with a vector in ${\mathbb {Q}}^{m(m-1)/2}$, this vector space can be endowed with the usual inner product.^10^

**Fig. 4:**
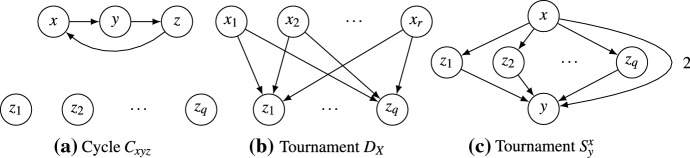
Some types of tournaments. Unlabelled arcs have weight 1

Let us define a few special weighted tournaments that will be useful later (see Fig. 4 for drawings). Given three distinct alternatives $x,y,z\in A$, we write $C_{xyz}$ for the weighted tournament with $[C_{xyz}]_{xy} = [C_{xyz}]_{yz} = [C_{xyz}]_{zx} = 1$, $[C_{xyz}]_{yx}= [C_{xyz}]_{zy} = [C_{xyz}]_{xz} = -1$, and all non-specified values 0. Thus, $C_{xyz}$ is a 3-cycle. Next, given a set $X\subseteq A$ of alternatives, we write $D_X$ for the weighted tournament with

$$\begin{aligned}{}[D_X]_{ab} = {\left\{ \begin{array}{ll} 1 &{} \text {if }\; a\in X, b \not \in X, \\ -1 &{} \text {if }\; a\not \in X, b \in X, \\ 0 &{} \text {otherwise}. \end{array}\right. } \end{aligned}$$

Thus, $D_X$ is the weighted tournament induced by a profile containing a singledichotomous voter *i* with $X \succ _i A{\setminus } X$. Finally, for alternatives $x,y\in A$ we will need the weighted tournament $S^x_y = D_{\{x\}} + D_{A{\setminus } \{y\}}$ which consists of a single “top” alternative *x*, a single “bottom” alternative *y*, and all other alternatives in between; *x* defeats *y* by a majority margin of 2.

The *cycle space*$V_\text {cycle}= \langle C_{xyz} :x,y,z\in A \text { distinct} \rangle$ is the subspace of *V* given by the span (the set of all linear combinations) of all 3-cycles $C_{xyz}$. It can also be written as the span of all simple cycles $C_{v_1v_2\dots v_k}$, since $C_{v_1v_2\dots v_k} = \sum _{i=2}^{k-1} C_{v_1v_iv_{i+1}}$. The *cocycle space*$V_\text {cocycle}= \langle D_{\{x\}} :x \in A \rangle$ is defined as the span of all tournaments $D_{\{x\}}$. It can also be written as the span of all $D_{X}$ with $X\subseteq A$, since $D_X = \sum _{x\in X} D_{\{x\}}$. For the tournaments in Fig. 2, we have that $T = C_{xyz}\in V_\text {cycle}$ and $T' = 4D_{\{x\}} + 2D_{\{y\}}\in V_\text {cocycle}$; the tournament $T''$ is neither contained in $V_\text {cycle}$ nor in $V_\text {cocycle}$.

As we now show, *V* can be decomposed as the sum $V = V_\text {cycle}+ V_\text {cocycle}$, and these two subspaces are orthogonal, i.e., for all $T_\text {cycle}\in V_\text {cycle}$ and $T_\text {cocycle}\in V_\text {cocycle}$, we have $T_\text {cycle}\cdot T_\text {cocycle}= 0$. This is a standard result, but we include a proof for completeness. The proof will also establish that $$\dim V_\text {cycle}= \left( {\begin{array}{c}m\\ 2\end{array}}\right) - (m - 1)$$ and $\dim V_\text {cocycle}= m-1$.

### Proposition 1

The subspaces $V_\text {cycle}$ and $V_\text {cocycle}$ are orthogonal and $V = V_\text {cycle}+ V_\text {cocycle}$.

### Proof

Since the inner product is bilinear, it suffices to check orthogonality on spanning sets of $V_\text {cycle}$ and $V_\text {cocycle}$. So consider some $C_{xyz}$ and some $D_{\{v\}}$ with $x,y,z,v\in A$. If $v\not \in \{x,y,z\}$, then $C_{xyz}\cdot D_{\{v\}} = 0$. Otherwise, without loss of generality, $v = x$ and so $C_{xyz}\cdot D_{\{x\}} = [C_{xyz}]_{xy}[D_{\{x\}}]_{xy} + [C_{xyz}]_{xz}[D_{\{x\}}]_{xz} = 1-1 = 0$.

To see that $V = V_\text {cycle}+ V_\text {cocycle}$, we use a dimension analysis. Write $A= \{ 1,\dots , m\}$. We claim that $D_{\{1\}}, \dots , D_{\{m-1\}}$ are linearly independent, so that $\dim V_\text {cocycle}\geqslant m-1$. Let $\lambda _1,\dots ,\lambda _{m-1}\in {\mathbb {Q}}$ be such that $\sum _{k = 1}^{m-1} \lambda _kD_{\{k\}} = 0$. Now, for all $i,j\in \{1,\dots ,m-1\}$,

$$\begin{aligned} \textstyle 0 = \left[ \sum _{k = 1}^{m-1} \lambda _kD_{\{k\}}\right] _{ij} = \lambda _i [D_{\{i\}}]_{ij} + \lambda _j [D_{\{j\}}]_{ij} = \lambda _i - \lambda _j. \end{aligned}$$

Also, $0 = [\sum _{i = k}^{m-1} \lambda _kD_{\{k\}}]_{1m} = \lambda _1[D_{\{1\}}] = \lambda _1$. Hence $\lambda _i = 0$ for all $i\in \{1,\dots ,m-1\}$.

We prove that $\dim V_\text {cycle}\geqslant {m\atopwithdelims ()2} - (m-1) = {m-1\atopwithdelims ()2}$ by showing that $\{C_{ijm}:i,j\in [m-1],\, i < j\}$ is a set of linearly independent vectors. For $i,j\in [m-1]$, $i < j$, let $\lambda _{ij}\in {\mathbb {Q}}$ be such that $\sum _{i=1}^{m-1}\sum _{j = i+1}^{m-1} \lambda _{ij} C_{ijm} = 0$. Hence, for all $i,j\in [m-1]$ with $i<j$,

$$\begin{aligned} \textstyle 0 = \left[ \sum _{k=1}^{m-1}\sum _{l = k+1}^{m-1} \lambda _{kl} C_{klm}\right] _{ij} = \lambda _{ij} [C_{ijm}]_{ij} = \lambda _{ij}. \end{aligned}$$

Thus, the $C_{ijm}$ are linearly independent. $\square$

With this decomposition, given a weighted tournament *T*, we can uniquely write $T = T_\text {cycle}+ T_\text {cocycle}$, where $T_\text {cycle}\in V_\text {cycle}$ is the *cyclic component* of *T* and $T_\text {cocycle}\in V_\text {cocycle}$ is the *cocyclic component* of *T*. We say that *T* is *purely cyclic* if $T = T_\text {cycle}$ so that $T_\text {cocycle}= 0$, and we say that *Tpurely cocyclic* if $T = T_\text {cocycle}$ so that $T_\text {cycle}= 0$. Of the examples in Fig. 4, $C_{xyz}$ is purely cyclic, and $D_X$ and $S^x_y$ are purely cocyclic. In Fig. 2, the tournament $T'' = T + T'$ can be decomposed into its cyclic component *T* and its cocyclic component $T'$.

In accordance with the definition for profiles, we define the Borda score $\beta _T(a)$ of an alternative *a* in a tournament $T \in V$ as

$$\begin{aligned} \beta _T(a) = \sum _{b\in A{\setminus }\{a\}} [T]_{ab}. \end{aligned}$$

If *T* is a purely cyclic tournament, then every alternative has Borda score 0. Hence the Borda scores induced by a given tournament *T* are the same as the Borda scores induced by its cocyclic component $T_\text {cocycle}$. In fact, knowledge of the Borda scores is enough to construct $T_\text {cocycle}$, as is shown by the following convenient characterization of purely cocyclic tournaments.

### Lemma 1

(Zwicker 2018) A weighted tournament *T* is purely cocyclic if and only if it is *difference generated*, i.e., there exists a function $\gamma :A \rightarrow {\mathbb {R}}$ such that $[T]_{xy}= \gamma (x) - \gamma (y)$ for all $x,y\in A$. In fact, if *T* is purely cocyclic, then it is difference generated by $\gamma (x) := \beta _T(x)/m$, i.e., by Borda scores, suitably rescaled.

The function $\gamma$ is unique up to adding a constant. We can normalize $\gamma$ by requiring that $\sum _{x\in A} \gamma (x) = 0$, in which case we then have $\gamma (x) = \beta _T(x)/m$ for all $x\in A$.

For example, the tournament $S^x_y$ is difference generated with $\gamma (x) = 1$, $\gamma (y) = -1$, and $\gamma (z) = 0$ for $z\in A {\setminus } \{x,y\}$. The tournament $D_{\{x\}}$ is difference generated with $\gamma (x) = (m-1)/m$ and $\gamma (z) = -1/m$ for all $z\in A{\setminus } \{x\}$.

### Proof of Lemma 1

If tournaments $T_1$ and $T_2$ are difference generated by $\gamma _1$ and $\gamma _2$, respectively, then it is easy to see that $\alpha _1 T_1 + \alpha _2 T_2$ is difference generated by $\alpha _1\gamma _1+ \alpha _2\gamma _2$. As we noted above, the tournaments $D_{\{x\}}$ are difference generated. Hence all tournaments in the space $V_\text {cocycle}$, which is spanned by the tournaments $D_{\{x\}}$, are difference generated.

Suppose tournament *T* is difference generated by $\gamma$, and consider the tournament $C_{xyz}$ for some $x,y,z\in A$. Then

$$\begin{aligned} T \cdot C_{xyz} = (\gamma (x) - \gamma (y)) + (\gamma (y) - \gamma (z)) + (\gamma (z) - \gamma (x)) = 0. \end{aligned}$$

Hence *T* is orthogonal to every $C_{xyz}$, and thus it is orthogonal to $V_\text {cycle}$, since $V_\text {cycle}$ is spanned by 3-cycles. Thus, by Proposition 1, *T* is purely cocyclic, i.e., $T\in V_\text {cocycle}$.

For the second statement, recall that $D_{\{x\}}$ is difference generated by $\gamma (x)= \beta _{D_{\{x\}}}(x)/m = (m-1)/m$ and $\gamma (z) = \beta _{D_{\{x\}}}(z)/m = -1/m$ for all $z\ne x$, since $\gamma (x) - \gamma (z) = \beta _{D_{\{x\}}}(x)/m - \beta _{D_{\{x\}}}(z)/m = 1$. Similarly to above, it then follows that a linear combination *T* of these tournaments is generated by $\gamma (x) = \beta _T(x)/m$. $\square$

With this result, it is easy to find the decomposition of a given tournament. First construct the cocyclic component $T_\text {cocycle}$ using the Borda scores, and then obtain $T_\text {cycle}= T - T_\text {cocycle}$.

## Characterization

We are now ready to state and prove our main result. For the remainder of this section, let ${\mathscr {D}}\subseteq {\mathscr {R}}$ be some fixed McGarvey domain.

### Theorem 1

A social dichotomy function *f* satisfies neutrality, reinforcement, the quasi-Condorcet property, and faithfulness if and only if *f* is the Borda mean rule.

The fact that the Borda mean rule satisfies all four axioms follows readily from the definition. Hence we only prove the “only if” part of Theorem 1. The proof is split up into five lemmas; in the statement of each lemma, we mention which axioms are used in its proof. Whenever cancellation suffices as a weakening of the quasi-Condorcet property, we note this as well.

Our first two lemmas are similar to lemmas in Young’s (1974a) characterization of Borda’s rule. Their conclusions do not depend on the type of output of the aggregation rule, and in particular also hold for social choice functions and social preference functions (for the appropriate definition of cancellation). Young (1974a) operates in the context of profiles of linear orders, but we can adapt the arguments to work for any McGarvey domain.

### Lemma 2

(Young 1974a; Young and Levenglick 1978) If a social dichotomyfunction *f* satisfies reinforcement and cancellation, then *f* only depends on themajority margins.

### Proof

Suppose *f* satisfies reinforcement and cancellation, and let $P_1$ and $P_2$ be two profiles that induce the same majority margins, i.e., $T(P_1) = T(P_2)$.

Assume first that $P_1$ and $P_2$ are defined on disjoint electorates. Let *Q* and $Q'$ be two profiles such that the electorates of all four profiles are pairwise disjoint and $T(Q) = T(P_1)$ and $T(Q') = - T(P_1\cup Q)$. The profile $Q'$ exists, since all majority margins in $P_1\cup Q$ are even (since $T(P_1\cup Q) = T(P_1) + T(Q) = 2\cdot T(P_1)$) and ${\mathscr {D}}$ is a McGarvey domain by assumption. Observe that $T(P_1\cup Q\cup Q') = T(P_2\cup Q\cup Q')= 0$. Thus, it follows from cancellation that $f(P_1\cup Q\cup Q') = f(P_2\cup Q\cup Q') = {\mathscr {R}}_2$. By reinforcement, we get

$$\begin{aligned} f(P_1) = f(P_1)\cap {\mathscr {R}}_2&= f(P_1)\cap f(P_2 \cup Q\cup Q') \\&= f(P_1\cup P_2 \cup Q\cup Q')\\&= f(P_1\cup Q\cup Q')\cap f(P_2) = {\mathscr {R}}_2\cap f(P_2) = f(P_2). \end{aligned}$$

For profiles $P_1$ and $P_2$ whose electorates are not disjoint, find a profile $P_3$ whose electorate is disjoint from both $P_1$ and $P_2$, and so that $P_3$ induces the same majority margins as $P_1$ and $P_2$. Using the argument above twice, we have $f(P_1) = f(P_3)= f(P_2)$. $\square$

A *tournament dichotomy function*$\varphi$ is a map from the set *V* of weighted tournaments to non-empty subsets of ${\mathscr {R}}_2$, so that $\varphi (T) \subseteq {\mathscr {R}}_2$ for all profiles *T*. A tournament dichotomy function $\varphi$*induces* a social dichotomy function *f* where $f(P) = \varphi (T(P))$ for all profiles $P\in {\mathscr {D}}^N$ with $N\in {\mathscr {F}}({\mathbb {N}})$.

Our axioms have natural analogues for tournament dichotomy functions $\varphi$.

Neutrality $$\begin{aligned} \varphi (\sigma (T)) = \sigma (\varphi (T))\quad \text {for all } T \in V \text {, and } \sigma \in \varPi (A). \end{aligned}$$

Reinforcement $$\begin{aligned} \varphi (T)\cap \varphi (T') \ne \varnothing \text { implies } \varphi (T)\cap \varphi (T') = \varphi (T + T') \quad \text {for all } T,T'\in V . \end{aligned}$$

Quasi-Condorcet property $$\begin{aligned}&{\succcurlyeq } \in \varphi (T) \text { if and only if } {\succcurlyeq '} \in \varphi (T)\quad \text {whenever }\succcurlyeq \text { and }\succcurlyeq '\text { agree on }A {\setminus } D, \\&\quad \text {where } D = \{x\in A:[T]_{xy} = 0\text { for all } y\in A\} \text {, for all } T\in V. \end{aligned}$$

Cancellation $$\begin{aligned}&\varphi (0) = {\mathscr {R}}_2. \end{aligned}$$

In the next lemma, we show that there is a one-to-one correspondence between tournament dichotomy functions and social dichotomy functions, when we restrict attention to functions satisfying reinforcement and cancellation.

### Lemma 3

(Young 1974a) Suppose the social dichotomy function *f* satisfiesreinforcement and cancellation. Then there exists a unique tournament dichotomy function $\varphi _f$ which induces *f* and satisfies reinforcement. Further, *f* satisfiesneutrality and the quasi-Condorcet property if and only if $\varphi _f$ does.

### Proof

Let *f* be a social dichotomy function satisfying reinforcement and cancellation. By Lemma 2, *f* only depends on the majority margins induced by a preference profile. Thus, *f* is *anonymous*, i.e., its outcome is invariant under renaming the voters. Let us observe that reinforcement and anonymity imply that *f* is *homogeneous*. Suppose $P\in {\mathscr {D}}^N$ and $P'\in {\mathscr {D}}^{N'}$ are profiles on disjoint electorates which are “copies”: there is a bijection $\xi :N \rightarrow N'$ with $P'(\xi (i)) = P(i)$ for all $i\in N$. By anonymity, $f(P) = f(P')$, and thus by reinforcement, $f(P \cup P') = f(P) \cap f(P') = f(P)$. By induction, if *nP* is a profile consisting of *n* copies of *P*, then $f(nP) = f(P)$.

We next construct a tournament dichotomy function $\varphi _f$ that induces *f*. Let $T\in V$ be a rational tournament. Take $n\in {\mathbb {N}}$ to be an integer such that *nT* is a tournament whose weights are even integers, i.e., such that $\frac{1}{2}nT\in {\mathbb {Z}}^{m\times m}$. Since ${\mathscr {D}}$ is a McGarvey domain, there is a preferences profile $P \in {\mathscr {D}}^N$ for some $N\in {\mathscr {F}}({\mathbb {N}})$ which induces *nT*. We define $\varphi _f(T) := f(P)$. Then $\varphi _f(T)$ is well-defined, that is, independent of the choice of *n* and *P*. To see this, suppose that *P* is a profile inducing *nT* and *Q* is a profile inducing $n'T$, for some $n,n'\in {\mathbb {N}}$. Then $T(n'P) = n\cdot n' \cdot T = T(nQ)$ and thus, because *f* is homogeneous and using Lemma 2, we have $f(P) = f(n'P) = f(nQ) = f(Q)$. Clearly, the resulting tournament dichotomy function $\varphi _f$ induces *f*.

Next, we show that $\varphi _f$ satisfies reinforcement. From the definition of $\varphi _f$, it follows immediately that $\varphi _f(nT) = \varphi _f(T)$ for all $T\in V$ and $n \in {\mathbb {N}}$. Let $T,T' \in V$ be such that $\varphi _f(T)\cap \varphi _f(T')\ne \varnothing$. Take $n\in {\mathbb {N}}$ with $\frac{1}{2}nT,\frac{1}{2}nT'\in {\mathbb {Z}}^{m\times m}$, and let $P,P'$ be profiles on disjoint electorates which induce *nT* and $nT'$, respectively. Note that $P \cup P'$ induces $nT + nT'$. By definition of $\varphi _f$, we also have $\varphi _f(T) = f(P)$ and $\varphi _f(T') = f(P')$ and thus, $f(P)\cap f(P')\ne \varnothing$. Then

$$\begin{aligned} \varphi _f(T+T')&= \varphi _f(n(T + T')) = \varphi _f(nT + nT') \\&= f(P \cup P') = f(P) \cap f(P') = \varphi _f(T) \cap \varphi _f(T'), \end{aligned}$$

because *f* satisfies reinforcement and $f(P) \cap f(P') \ne \varnothing$.

To see uniqueness, let $\varphi$ be any tournament dichotomy function that satisfiesreinforcement and induces *f* so that $\varphi (T(P)) = f(P)$ for all profiles *P*. We show that $\varphi = \varphi _f$. To this end, let $T \in V$, and take $n \in {\mathbb {N}}$ with $\frac{1}{2}nT\in {\mathbb {Z}}^{m\times m}$ and a profile *P* inducing *nT*. Then

$$\begin{aligned} \varphi (T) = \varphi (nT) = f(P) = \varphi _f(T), \end{aligned}$$

where the first equality follows because $\varphi (T + T) = \varphi (T)$ by reinforcement.

The fact that *f* satisfies neutrality and the quasi-Condorcet property if and only if $\varphi _f$ does follows easily from the definition of $\varphi _f$. $\square$

Based on Lemma 3, for a social dichotomy function *f* satisfying reinforcement and cancellation, we write $\varphi _f$ for the unique tournament dichotomy function that induces *f* and satisfies reinforcement. For example, $\varphi _ BM$ denotes the tournament dichotomy function induced by the Borda mean rule.

The rest of the argument will focus on tournament dichotomy functions. We will prove that if $\varphi$ satisfies neutrality, reinforcement, and the quasi-Condorcet property, then $\varphi \in \{\varphi _ BM ,\varphi _{- BM },\varphi _ TRIV \}$. Lemma 3 will then allow us to reach the analogous conclusion about the social dichotomy function *f*.

Our next lemmas show that $\varphi$ only depends on the Borda scores of the alternatives. This is done by showing that $\varphi$ is trivial on purely cyclic tournaments, in the sense of returning all dichotomies. It follows from reinforcement that the cyclic part of a weighted tournament can be ignored when computing the outcome of $\varphi$. Since the cocyclic part is completely determined by the Borda scores (see Lemma 1), $\varphi$ can only depend on the Borda scores.

As a first step, we show that $\varphi$ is trivial for the building blocks $C_{xyz}$ of the cycle space, by an argument using neutrality.

### Lemma 4

If a tournament dichotomy function $\varphi$ satisfies neutrality, reinforcement, and cancellation, then $\varphi (C_{xyz}) = {\mathscr {R}}_2$ for all distinct $x,y,z\in A$.

### Proof

Let $x,y,z\in A$ be three distinct alternatives, write $C = C_{xyz}$, and take any${\succcurlyeq }\in \varphi (C)$. Since $\succcurlyeq$ is dichotomous, it cannot be that *x*, *y*, *z* are all in distinct indifference classes, and so $\succcurlyeq$ is indifferent between at least two of them. Hence, we may assume without loss of generality that $x\sim y$. Let $\sigma = (x \, y)$ be the permutation that transposes *x* and *y* and leaves other alternatives fixed. We have ${\succcurlyeq } = \sigma ({\succcurlyeq }) \in \sigma (\varphi (P)) = \varphi (\sigma (C))$, where the last equality follows from neutrality. Next, note that $\sigma (C) = C_{zyx}$ which is the reverse of *C*. Hence, $C + \sigma (C) = 0$. Since ${\succcurlyeq } \in \varphi (C) \cap \varphi (\sigma (C))$, by reinforcement,

$$\begin{aligned} \varphi (C) \cap \varphi (\sigma (C)) = \varphi (C + \sigma (C)) = \varphi (0) = {\mathscr {R}}_2, \end{aligned}$$

where the last step follows from cancellation. Hence $\varphi (C) = {\mathscr {R}}_2$. $\square$

Next, we lift the result for 3-cycles $C_{xyz}$ to apply to all tournaments in $V_\text {cycle}$.

### Corollary 1

If a tournament dichotomy function $\varphi$ satisfies neutrality, reinforcement, and cancellation, then $\varphi$ depends only on Borda scores.

### Proof

Let *T* be any weighted tournament, and consider its orthogonal decomposition $T = T_{\text {cycle}} + T_{\text {cocycle}}$. We will show that $\varphi (T) = \varphi (T_\text {cocycle})$. Since $T_\text {cocycle}$ only depends on Borda scores (by Lemma 1), then so does $\varphi$. As the space of purely cyclic tournaments is spanned by 3-cycles, we can write

$$\begin{aligned} T_\text {cycle}= \sum _{\begin{array}{c} x,y,z\in A \\ \text {distinct} \end{array}} \lambda _{xyz} C_{xyz}, \end{aligned}$$

for some $\lambda _{xyz} \in {\mathbb {Q}}$, where we may assume $\lambda _{xyz} \geqslant 0$ for all $x,y,z\in A$ because we can replace negative values by observing that $C_{xyz} = -C_{zyx}$. By Lemma 4, we have $\varphi (C_{xyz}) = {\mathscr {R}}_2$ for all distinct $x,y,z \in A$. Thus, by reinforcement,

$$\begin{aligned} \varphi (T_\text {cycle}) = \bigcap _{\lambda _{xyz} > 0} \varphi (C_{xyz}) = {\mathscr {R}}_2. \end{aligned}$$

Thus, again by reinforcement, $\varphi (T) = \varphi (T_\text {cycle}) \cap \varphi (T_\text {cocycle}) = \varphi (T_\text {cocycle})$. $\square$

With the conclusion of Corollary 1 in place, the quasi-Condorcet property becomes a much stronger axiom: while previously it only implied that dummy alternatives (those that are majority-tied with every other alternative) can be moved around freely, now we see that this is the case for all alternatives with Borda score 0. Note that dummy alternatives have Borda score 0. Also, for every weighted tournament *T*, there is a weighted tournament $T'$ such that each alternative has the same Borda score in *T* and $T'$, and every alternative with Borda score 0 is a dummy alternative in $T'$. The tournament $T'$ can be constructed by subtracting a purely cyclic tournament from *T* that contains exactly those arcs in *T* that are adjacent to some alternative with Borda score 0. Since *T* and $T'$ have the same Borda scores and $\varphi$ only depends on Borda scores, $\varphi$ must treat dummy alternatives and alternatives with Borda score 0 identically.

Next we observe that $\varphi$ is equivalent to the Borda mean rule for the purely cocyclic tournaments $S^x_y$ shown in Fig. 4c. These tournaments have the useful property that the Borda score of all but two alternatives is zero, and, as we will see in the proof of Lemma 6, every purely cocyclic tournament can be decomposed into such tournaments. The proof of Lemma 5 is the only place where we use the full force of the quasi-Condorcet property rather than only cancellation.

### Lemma 5

If a tournament dichotomy function $\varphi$ satisfies neutrality, reinforcement, and the quasi-Condorcet property, then $\varphi (S^x_y) \in \{\varphi _ BM (S^x_y),\varphi _{- BM }(S^x_y),{\mathscr {R}}_2\}$ for all $x,y\in A$.

**Fig. 5:**
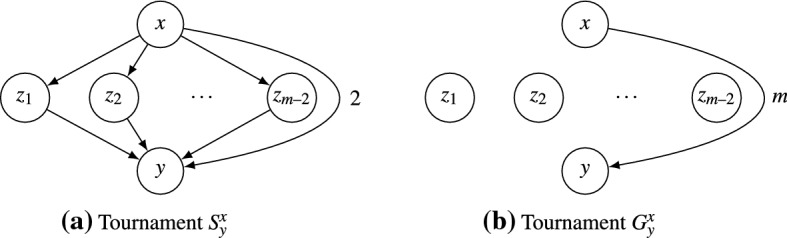
The tournaments used in the proof of Lemma 5. Each alternative has the same Borda score in either tournament: $\beta _{S^x_y}(x) = \beta _{G^x_y}(x) = m$, $\beta _{S^x_y}(y) = \beta _{G^x_y}(y) = -m$, and $\beta _{S^x_y}(z_i) = \beta _{G^x_y}(z_i) = 0$ for all $i\in \{1,\dots ,m-2\}$

### Proof

Recall that $|A| = m$. Let $G^x_y$ be the weighted tournament given by

$$\begin{aligned}{}[G^x_y]_{xy} = m, \quad [G^x_y]_{yx} = -m, \quad \text {and } [G^x_y]_{zv} = 0 \text { for }z,v\in A {\setminus } \{x,y\}. \end{aligned}$$

For each alternative, its Borda score in $S^x_y$ is equal to its Borda score in $G^x_y$ (see Fig. 5). By Corollary 1, $\varphi (G^x_y) = \varphi (S^x_y)$, and so it suffices to show that $\varphi (G^x_y)\in \{\varphi _ BM (G^x_y),\varphi _{- BM }(G^x_y),{\mathscr {R}}_2\}$. Note that $\varphi _ BM (G^x_y) = \varphi _ BM (S^x_y) = \{ {\succcurlyeq } \in {\mathscr {R}}_2 :x \succ y \}$.

Suppose first that there is ${\succcurlyeq }\in \varphi (G^x_y)$ such that $x\sim y$. Neutrality applied to the permutation $\sigma = (x\, y)$ implies that ${\succcurlyeq }\in \varphi (\sigma (G^x_y)) = \varphi (G^y_x)$. Hence, $\varphi (G^x_y)\cap \varphi (G^y_x)\ne \varnothing$, so reinforcement and cancellation imply that

$$\begin{aligned} \varphi (G^x_y)\cap \varphi (G^y_x) = \varphi (G^x_y + G^y_x) = \varphi (0) = {\mathscr {R}}_2. \end{aligned}$$

Hence $\varphi (G^x_y) = {\mathscr {R}}_2$, and we are done.

In $G^x_y$, all alternatives except *x* and *y* are dummies. Thus, if there is ${\succcurlyeq }\in \varphi (G^x_y)$ such that $x\succ y$, then by the quasi-Condorcet property, $\varphi _ BM (G^x_y) = \{{\succcurlyeq }\in {\mathscr {R}}_2:x\succ y\}\subseteq \varphi (G^x_y)$. Similarly, if there is ${\succcurlyeq }\in \varphi (G^x_y)$ such that $y\succ x$, then $\varphi _{- BM }(G^x_y) \subseteq \varphi (G^x_y)$.

Suppose next that there are ${\succcurlyeq },{\succcurlyeq '}\in \varphi (G^x_y)$ such that $x\succ y$ and $y\succ ' x$. Then $\varphi _ BM (G^x_y) \cup \varphi _{- BM }(G^x_y) \subseteq \varphi (G^x_y)$. Neutrality applied to the permutation $\sigma = (x\,y)$ implies

$$\begin{aligned} \varphi _{- BM }(G^x_y) \cup \varphi _ BM (G^x_y) = \sigma (\varphi _ BM (G^x_y)) \cup \sigma (\varphi _{- BM }(G^x_y)) \subseteq \varphi (\sigma (G^x_y)) = \varphi (G^y_x). \end{aligned}$$

Thus, $\varphi (G^x_y)\cap \varphi (G^y_x)\ne \varnothing$, so reinforcement and cancellation imply that

$$\begin{aligned} \varphi (G^x_y)\cap \varphi (G^y_x) = \varphi (G^x_y + G^y_x) = \varphi (0) = {\mathscr {R}}_2. \end{aligned}$$

Hence $\varphi (G^x_y) = {\mathscr {R}}_2$, and we are done.

In the remaining case, either $x \succ y$ for all ${\succcurlyeq } \in \varphi (G^x_y)$ and hence $\varphi (G^x_y)= \varphi _ BM (G^x_y)$; or $y \succ x$ for all ${\succcurlyeq } \in \varphi (G^x_y)$ and hence $\varphi (G^x_y) = \varphi _{- BM }(G^x_y)$. $\square$

In combination with our axioms, either one of the three cases characterized in Lemma 5 pins down $\varphi$ on all tournaments.

### Lemma 6

If a tournament dichotomy function $\varphi$ satisfies neutrality, reinforcement, and the quasi-Condorcet property, then $\varphi \in \{\varphi _ BM ,\varphi _{- BM },\varphi _ TRIV \}$.

### Proof

Take any distinct $x,y\in A$ and choose $\psi \in \{\varphi _ BM ,\varphi _{- BM },\varphi _ TRIV \}$ so that $\varphi (S^x_y) = \psi (S^x_y)$. By Lemma 5, the choice of $\psi$ is well-defined, unique, and, by neutrality of $\varphi$, independent of *x*, *y*.

We will show that $\varphi = \psi$. By Corollary 1, it suffices to show that $\varphi$ is equal to $\psi$ for purely cocyclic tournaments. Let *T* be a purely cocyclic tournament. We prove the statement by induction on the number of alternatives with non-zero Borda score in *T*.

If there are no such alternatives, i.e., every alternative has Borda score 0, then $T=0$ since *T* is purely cocyclic. By cancellation (implied by the quasi-Condorcet property), we have $\varphi (T) = {\mathscr {R}}_2 = \psi (T)$.

Now assume that not all alternatives have Borda score 0 in *T*, and that $\varphi (T')= \psi (T')$ for all $T'\in V$ in which fewer alternatives have non-zero Borda score than in *T*. By Lemma 1, *T* is difference generated by the function $\gamma :A\rightarrow {\mathbb {R}}$, $\gamma (a)= \beta _T(a)/m$. Since not all Borda scores are 0, $\gamma$ is not constant. Let ${\bar{x}} \in \mathop {\hbox {arg max}}\limits \nolimits _{x\in A} \gamma (x)$ and $\underline{x} \in \mathop {\hbox {arg min}}\limits \nolimits _{x\in A} \gamma (x)$. Since $\sum _{x\in A} \gamma (x) = 0$, we have $\gamma ({\bar{x}}) > 0$ and $\gamma (\underline{x}) < 0$. Let $\delta = \min \{|\gamma ({\bar{x}})|, |\gamma (\underline{x})|\} > 0$. Let ${\hat{T}}$ be the tournament that isdifference generated by ${\hat{\gamma }}:A\rightarrow {\mathbb {R}}$ with

$$\begin{aligned} {\hat{\gamma }}({\bar{x}}) = \gamma ({\bar{x}}) - \delta \text {, } {\hat{\gamma }}(\underline{x}) = \gamma (\underline{x}) + \delta \text {, and } {\hat{\gamma }}(x) = \gamma (x) \text { for all }x\in A{\setminus }\{{\bar{x}}, \underline{x}\}. \end{aligned}$$

Now $\sum _{x\in A} {\hat{\gamma }}(x) = 0$, and so we have that ${\hat{\gamma }}(a) = \beta _{{\hat{T}}}(a)/m$ for all $a\in A$ (see the remark after Lemma 1). Note that either ${\bar{x}}$ or $\underline{x}$ now has Borda score 0 in ${\hat{T}}$. Thus, there are fewer alternatives with non-zero Borda score in ${\hat{T}}$ than in *T*, and so $\varphi ({\hat{T}}) = \psi ({\hat{T}})$ by the inductive hypothesis. Also, $\varphi (S^{{\bar{x}}}_{\underline{x}}) = \psi (S^{{\bar{x}}}_{\underline{x}})$, by definition of $\psi$. For each of the three possible choices of $\psi$, we have $\psi ({\hat{T}})\cap \psi (S^{{\bar{x}}}_{\underline{x}})\ne \varnothing$. From this and $T = {\hat{T}} + \delta \smash {S_{\underline{x}}^{{\bar{x}}}}$, it follows by reinforcement of $\varphi$ and $\psi$ that

$$\begin{aligned} \varphi (T) = \varphi ({\hat{T}}) \cap \varphi (S_{\underline{x}}^{{\bar{x}}}) = \psi ({\hat{T}}) \cap \psi (S_{\underline{x}}^{{\bar{x}}}) = \psi (T), \end{aligned}$$

which completes the proof. $\square$

Combining the results of our lemmas, our main result follows.

### Proof of Theorem 1

Suppose *f* is a social dichotomy function satisfying neutrality, reinforcement, and the quasi-Condorcet property. By Lemma 3, the tournament dichotomy function $\varphi _f$ inducing *f* also satisfies neutrality, reinforcement, and the quasi-Condorcet property. Thus, by Lemma  6, we have $\varphi _f \in \{\varphi _ BM ,\varphi _{- BM },\varphi _ TRIV \}$. From the uniqueness part of Lemma 3, it follows that $f\in \{ BM ,- BM , TRIV \}$.

If *f* further satisfies faithfulness, then $$f = BM $$, since neither $$-{ BM }$$ nor $$ TRIV $$ satisfies faithfulness. To see this, consider $$P\in {\mathscr {D}}^{\{i\}}$$, $$i\in {\mathbb {N}}$$, such that $$\succcurlyeq _i$$ is not complete indifference, which exists since $${\mathscr {D}}$$ is a McGarvey domain. Let $$x,y\in A$$ be such that *x* is most-preferred and *y* is least-preferred for *i*; that is, $$x \succcurlyeq _i z \succcurlyeq _i y$$ for all $$z\in A$$. Then $$x \succ _i y$$, and it is easy to check that $$\beta _P(x)> 0 > \beta _P(y)$$. Faithfulness requires that if $${\succcurlyeq } \in f(P)$$, then $${\succ _i} \subseteq {\succcurlyeq }$$; in particular, $$x \succcurlyeq y$$. However, by their definitions, both $$ TRIV (P)$$ and $$- BM (P)$$ contain dichotomies with $$y \succ x$$. $$\square $$

## Independence of the axioms

We show that all four axioms are indeed required for the characterization by giving a social dichotomy function that satisfies all but one of the axioms for each of the four axioms.

- *Neutrality:* Fix two alternatives $a,b\in A$ and define a skewed variant of the Borda mean rule, which computes the tournament induced by the input profile, doubles the weight of the arc between *a* and *b*, and then calculates the outcome of the Borda mean rule.
- *Reinforcement:* Apply the $\mathrm {sign}$-function to all majority margins (i.e., replacepositive numbers by $+\,1$ and replace negative numbers by $-\,1$) beforecalculating the outcome of the Borda mean rule. This yields the *Copeland mean rule* that approves all alternatives with above-average Copeland score anddisapproves those with below-average Copeland score.
- *Faithfulness:* Reverse all dichotomous weak orders returned by the Borda mean rule ($$- BM $$) or always return all dichotomies ($$ TRIV $$). Lemma 6 shows that these are in fact the only other social dichotomy functions that satisfy the remaining axioms.
- *Quasi-Condorcet property:* Whenever all alternatives have Borda score zero (the weighted tournament is purely cyclic) then return all dichotomies. Otherwise, return the Borda winners, in the sense of returning $\{ D_{\{x\}} :x \text { is a Borda winner} \}$. By case analysis, one can check that this rule satisfies reinforcement. Notice that it does not satisfy reversal symmetry.

The last example implies that, in our main result, we cannot weaken the quasi-Condorcet property to cancellation.

## Conclusions and future work

We have presented a characterization of the Borda mean rule as a social dichotomy function, showing that it fills the same space as does Kemeny’s rule among social preference functions. It would be interesting to see other social dichotomy functions discussed in the literature. For example, one might consider mean rules based on other positional scoring rules. Lang et al. (2016) propose a version of the Ranked Pairs rule that returns dichotomies, and Kilgour (2016) proposes some multiwinner voting rules with committees of variable size, which can be interpreted as social dichotomy functions. For now, the Borda mean rule seems like a very attractive example of a social dichotomy function.

Several questions remain for future work. Is there an alternative proof of ourcharacterization that does not need linear algebra, such as in the proof of Hansson and Sahlquist (1976) for Borda’s rule and of Debord (1992) for the *k*-Borda rule? We can also ask whether the Borda mean rule can be characterized using different axioms. It seems particularly desirable to replace the quasi-Condorcet property with a more intuitive axiom. For example, does our result still hold if we were to replace thequasi-Condorcet property with the conjunction of cancellation and reversalsymmetry? Or if we replace it with cancellation together with the requirement that Condorcet winners are always approved and Condorcet losers are always disapproved? These results are not ruled out by our examples in Sect. 8; to establish them, one would only need to reprove the conclusion of Lemma 5.

The Borda mean rule is particularly natural if voters’ preferences are themselves dichotomous; in this setting, the Borda mean rule is called the *mean rule* (Duddy et al. 2016). Our proof does not characterize the Borda mean rule if it is defined only over dichotomous preference profiles, because the quasi-Condorcet property is equivalent to cancellation on this domain. It would be interesting to have an axiomatic characterization of the mean rule using reinforcement. In a different formal setting, an axiomatic characterization using another consistency notion is already known (Duddy et al. 2016).

We have noted that the Borda mean rule can also be seen as the 2-Kemeny rule. It seems plausible that our axioms in fact also characterize the *k*-Kemeny rule for each $k\geqslant 3$. However, it seems that different techniques (closer to the ones employed by Young and Levenglick (1978)) are necessary to show this.

Finally, is there a similar characterization of *scoring mean rules* based on other scoring rules, in the style of Young (1975)?

## Footnotes

1 This example was suggested to us by Klaus Nehring.

2 Zwicker requires *k*-chotomous weak orders to have *exactly**k* indifference classes. This distinction will not be critical for our paper (see Footnote 5).

3 For expository purposes, Young and Levenglick (1978) introduce what they call the “Condorcet axiom” which is a substantial strengthening of faithfulness and the quasi-Condorcet property. However, as they note, this strengthening is not used or required in their proof.

4 Similar decompositions have been explored earlier by Harary (1959, 1969) and Croom (1978).

5 One could require that the output of a social dichotomy function never contains the weak order denoting complete indifference. All of our arguments remain valid for this alternative definition.

6 A matrix $M\in {\mathbb {R}}^{m\times m}$ is skew-symmetric if $M = -M^T$.

7 In fact, it is sufficient for our proof that the domain ${\mathscr {D}}$ is rich enough to induce all tournaments with *even* entries.

8 Ours is not the only sensible extension of Borda scores to weak orders. An alternative choice for the score for $a\in A$ would be $\sum _{i\in N} (m - {\text {rank}}_i(a))$, where ${\text {rank}}_i(a)) = k$ iff *a* is in the *k*th highest indifference class of $\succcurlyeq _i$. This alternative definition notably cannot be written only in terms of the majority margins $[T(P)]_{ab}$. The two definitions are discussed by Chebotarev and Shamis (1998, Sect. 3.4) and Gärdenfors (1973, Sect. 7).

9 Adapting Fishburn’s (1977) classification of the informational requirements of social choice functions to social dichotomy functions (whose input could contain weak orders), the Borda mean rule is a *C2 rule*.

10 The inner product of two vectors $u, v \in {\mathbb {Q}}^n$ is defined to be $u \cdot v = \sum _{i=1}^n u_iv_i$.
